# Improvement of the detection efficiency calibration and homogeneity measurement of Si-SPAD detectors

**DOI:** 10.1186/s40064-016-3735-7

**Published:** 2016-12-01

**Authors:** Klodian Dhoska, Helmuth Hofer, Beatrice Rodiek, Marco López, Toomas Kübarsepp, Stefan Kück

**Affiliations:** 1 Tallinn University of Technology, Ehitajate tee 5, 19086 Tallinn, Estonia; 2 Physikalisch-Technische Bundesanstalt (PTB), Bundesallee 100, 38116 Brunswick, Germany; 3 AS Metrosert, Teaduspargi 8, 12618 Tallinn, Estonia

**Keywords:** Detection efficiency, Si-SPAD detector, Alignment, Integrating sphere, Homogeneity

## Abstract

**Background:**

Silicon single-photon avalanche diodes (Si-SPADs) are the most used devices for measuring ultra-weak optical radiant fluxes in many quantum technology fields, such as quantum optics, quantum communication, quantum computing, etc. In all these fields, the detection efficiency is the main parameter, which has to be accurately known for achieving reliable measurements. In this paper we present the improvements performed on the setup described in López et al. (J Mod Opt 62:S21–S27, 2015) for determining the detection efficiency of Si-SPAD detectors with a low measurement uncertainty. The improvement arises from the precise alignment of the Si-SPAD detector and the low deviation reached between the total calculated filter transmission and the individual filter transmission measurements (≤0.05%) performed with an integrating sphere with attached Si-photodiode as standard detector.

**Results:**

The relative standard uncertainty of the Si-SPAD detection efficiency measurement achieved is now as low as ~0.16%. Furthermore, the investigation of the detection efficiency homogeneity of two commercial Si-SPAD detectors from different manufacturers and with different sensor diameters is also presented. The obtained homogeneity is ≤2.2% within a region of diameter of 40 μm.

**Conclusions:**

The detailed analysis presented in this paper shows the potential for achieving low measurement uncertainties for Si-SPAD detector calibration even in the low photon flux range. The low uncertainties are only to be realized for reproducible measurement conditions, i.e. in specific for equal beam sizes and beam shapes and well as for an irradiation of equal active areas of the detector. This, however, will be difficult to obtain when measurements are performed at different national metrology institutes.

## Background

Nowadays, Silicon single-photon avalanche diodes (Si-SPADs) are gaining more and more importance in a variety of different quantum technology fields, i.e. experimental quantum optics, quantum cryptography, quantum computing, as well as in medicine, 3D-imaging, biology, telecommunications and astrophysics (Knill et al. 2001; O’Brien 2007; O’Brien et al. 2009). In all these fields, the detection efficiency is a key parameter required to efficiently measure the optical radiant flux at photon levels; i.e. the detection efficiency must be determined using standard detectors or procedures that are traceable to primary reference standards.

There are two approaches mostly used for determining the detection efficiency of Si-SPAD detectors: one is based on the detector subtitution technique, which uses a strongly attenuated laser and a reference detector (López et al. 2015; Dhoska et al. 2015a, b; Kück et al. 2014) and the other one based on the two-photon correlation technique (Polyakov et al. 2006). The latter has the advantage that it does not require a reference detector; however, the effect of multiple photon events at photon-counting level has to be considered. The lowest uncertainty so far reported using this approach is 0.18%. Nevertheless, even when the achieved uncertainty is similar to those reported using the detector substitution technique (*u* = 0.16–0.3%) (López et al. 2015; Müller et al. 2012), the latter is mostly preferred by most of the national metrology institutes, since it uses a calibrated reference detector traceable to the primary reference standard (cryogenic radiometer) for the optical radiant power measurement; and thus, the traceability to a national primary standard is in this way fully assured. For this reason, the setup used to determine the detection efficiency of Si-SPAD detectors at Physikalisch-Technische Bundesanstalt (PTB), the German National Metrology Institute, is based on this approach, which uses the double attenuator technique (López et al. 2015). In this case, a standard detector (Si-Photodiode) is used to calibrate two attenuators required to attenuate the laser radiant power impinging on the Si-SPAD detector. These measurements are performed in situ subsequently; thus, the total attenuation is calculated by multiplying the two attenuation values. Knowing the total filter attenuation, the total optical power impinging on the Si-SPAD detector can be calculated and compared with the count rate generated by the Si-SPAD detector. From these measurements the detection efficiency of the Si-SPAD detector is determined. Using this measurement procedure, the detection efficiency was determined with a relative standard uncertainty of approx. 0.3% (López et al. 2015). The major uncertainty contribution to this measurement arose from the uncertainty associated to the measurement of the filter transmission, which is obtained from the deviation between the individual and combined transmission measurement carried out with an analog standard Si-photodiode (López et al. 2015; Dhoska et al. 2015a, b; Kück et al. 2014).

In this paper, we present the recent improvements carried out to this setup for achieving a low uncertainty for the Si-SPAD detection efficiency calibration. These consist in improving the filter transmission measurement and in implementing an accurate and automatic alignment procedure of the Si-SPAD detector. Furthermore, the mapping of the quantum detection efficiency homogeneity is also presented.

## Measurement setup

**Fig. 1:**
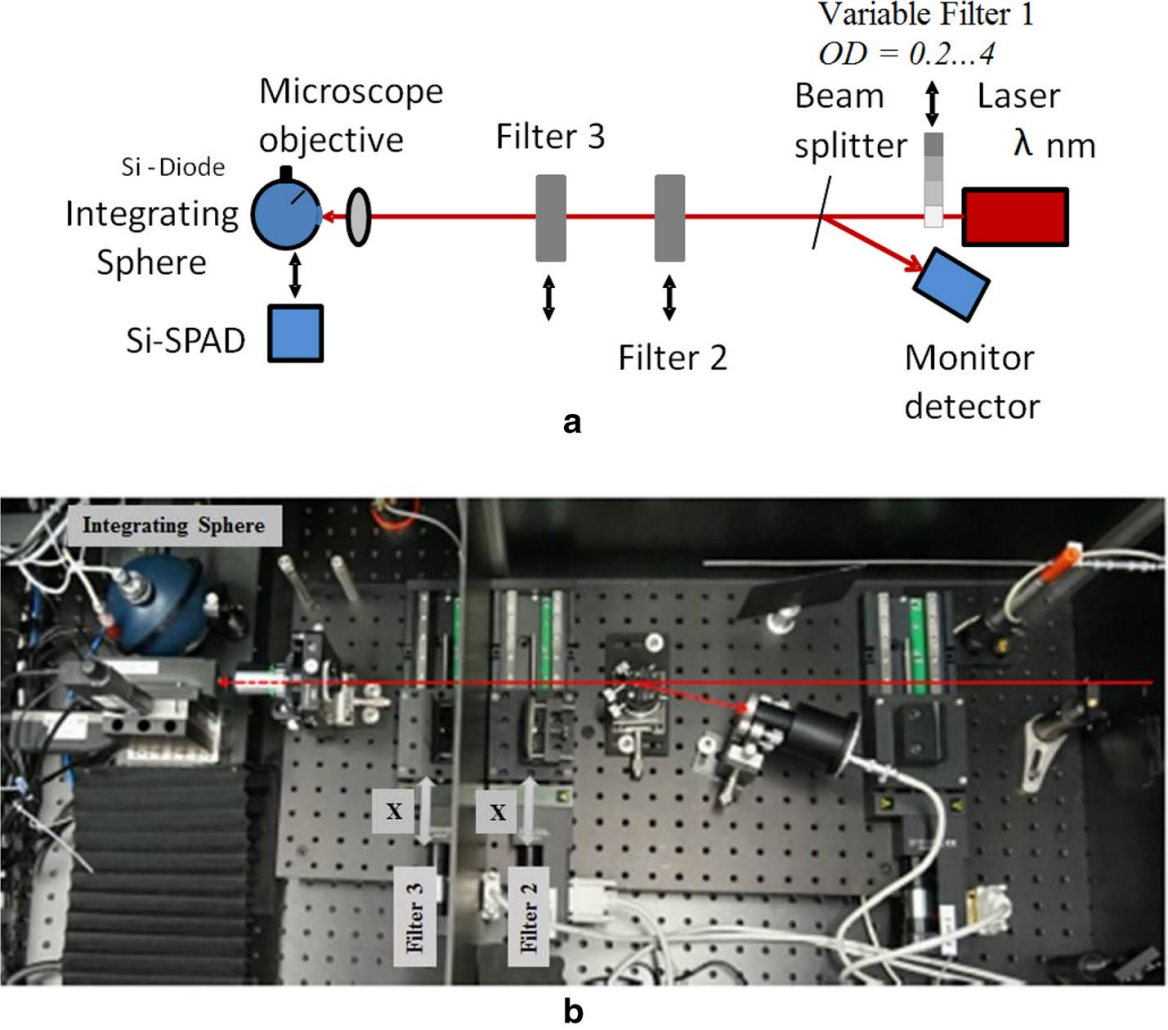
**a** Scheme and **b** photograph of the setup used for the calibration of the Si-SPAD detection efficiency by using an integrating sphere with an attached Si-photodiode as a reference standard detector. Si-photodiode: Hamamatsu S1227 66BR, Integrating sphere: Labsphere IS 40, Objective: Mitutoyo M-Plan Apo 20×, Tunable laser: New Focus 6312)

Figure 1a, b show the improved measurement setup and its photograph, respectively. Unlike in López et al. (2015), in this case an integrating sphere (Labsphere IS40) with attached Si-photodiode (Hamamatsu S1227 66BR) is used as a standard detector for measuring the optical flux as well as the filter transmission. The Si-photodiode is operated in the short-circuited mode (without bias voltage), and the photocurrent is converted to voltage by a trans-impedance amplifier (Gigahertz-Optik P-9202-4). During the Si-SPAD calibration procedure, the laser beam is focused onto the SPAD detector active area and into the integrating sphere by using a microscope objective lens (magnification *X* = 20, numerical aperture *NA* = 0.42, working distance *d*_w_ = 20 mm). The optimal alignment position of the Si-SPAD detector is located at the focal plane (working distance) of the objective lens, since at this position the active area of the Si-SPAD detector is underfilled by the laser beam. This diminishes the influence of detection efficiency inhomogeneities. Moreover, a monitor detector (Si, Photodiode, Hamamatsu S1227 1010BQ) is used to reduce possible fluctuations of the laser optical power during the measurements. The monitor detector is operated in a short-circuit mode, and a trans-impedance amplifier is used to convert the photocurrent into a voltage.

Two different types of Si-SPAD detectors with different sensor diameters were used in the experiments: a Single Photon Counting Module (SPCM) (Perkin-Elmer SPCM-AQR) with a sensor diameter of *ϕ*_D1_ = 180 μm (http://www.pas.rochester.edu/~advlab/APD_SPCM_AQR.pdf) and a Si-SPAD (Micro Photon Device PDM) with a sensor diameter of *ϕ*_D2_ = 50 μm (http://www.micro-photon-devices.com/Docs/Datasheet/PDM.pdf). The operating temperature of the Si-SPAD detectors was 24 °C.

### Si-SPAD alignment procedure

**Fig. 2:**
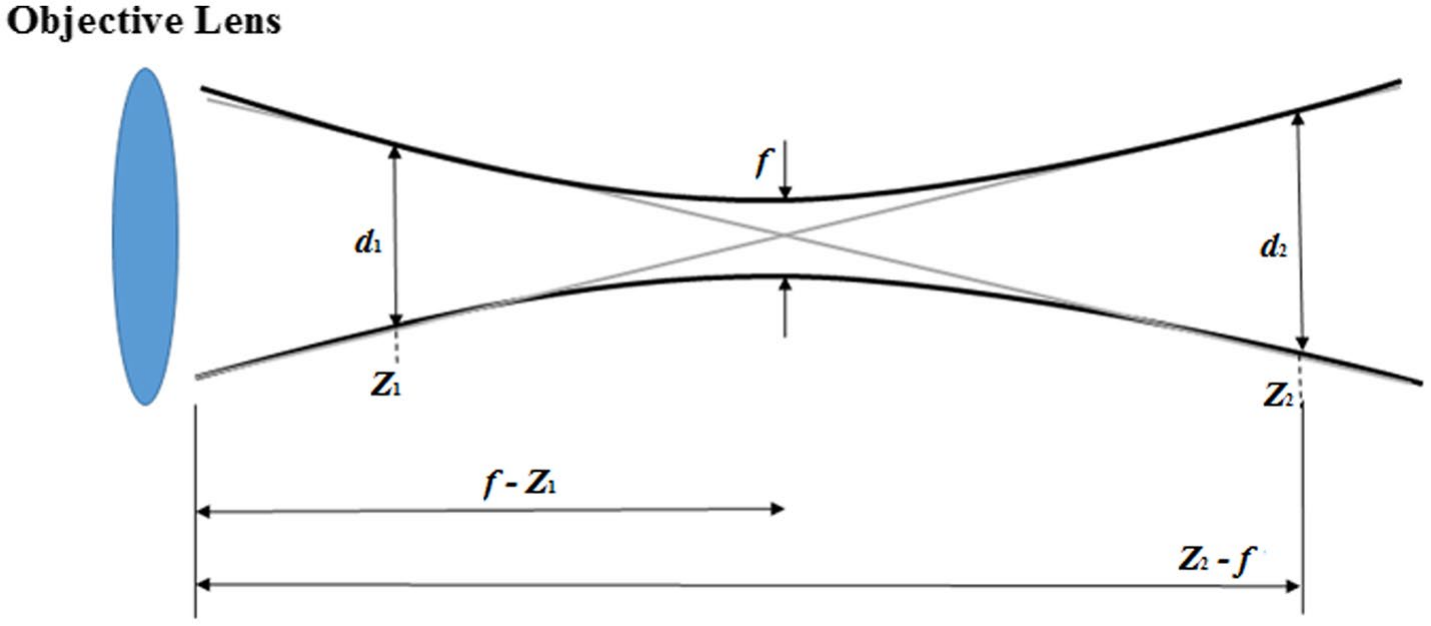
Schematic view of the beam propagation used for determining the objective focal plane. *f* is objective focal plane, *d*_1_ and *d*_2_ are beam diameters and *Z*_1_ and *Z*_2_ are positions where the two scans are performed for calculating the focal plane position *f*

The alignment of the Si-SPAD detector with respect to the focused beam is performed using motorized XYZ-translation stages in an automated manner. This is carried out in three steps: First, two *xy*-scans are performed using the Si-SPAD itself, one scan in front and the other behind the objective front focal plane. Although the absolute location of the focal plane is not exactly known, it can be roughly estimated, so that the scan positions for these two scans are easily found. In any case, these positions should be far away from the focal plane, so that the active area of the Si-SPAD detector is much smaller than the laser beam profile. As a result, these two scans will reveal mainly the laser beam profiles, i.e. Gaussian beam profiles. Second, the geometric parameters of these two beam profiles, i.e. the beam diameters and the centre positions are calculated by means of a Gaussian model fitting (or, as will be seen below, by a computing algorithm as the one described in the third step). Using this information, the focal length *f* of the microscope objective is calculated by approximating the beam profile to a simple geometric beam propagation, see dashed line in Fig. 2:

1 $$f = \frac{{d_{1} \cdot Z_{2} + d_{2} \cdot Z_{1} }}{{d_{1} + d_{2} }},$$

where *f* is the objective focal plane, *Z*_1_, *Z*_2_ are the scan positions in the *z*-axis and *d*_1_, *d*_2_ are the beam profile diameters, respectively. Third, a *xy*-scan is performed at the calculated focus position. In this case the obtained scan profile corresponds to a rectangular distribution, because here the active area of the Si-SPAD detector is much larger than the laser beam spot. The centre of the rectangular profile is calculated using the centroid algorithm (Neal et al. 2004), expressed below for *x*- and *y*-coordinates, respectively:

2 $$x_{center} = \frac{{\sum\nolimits_{i = 1}^{N} {x_{i} \cdot s_{i} } }}{{\sum\nolimits_{i = 1}^{N} {s_{i} } }},$$

3 $$y_{center} = \frac{{\sum\nolimits_{i = 1}^{N} {y_{i} \cdot s_{i} } }}{{\sum\nolimits_{i = 1}^{N} {s_{i} } }},$$

where *s*_i_ are the detector signals and *x*_i_ *, y*_i_ are the scanning positions in *x*- and *y*-coordinates. The diameters of each rectangular beam profile (*x*_diameter_ and *y*_diameter_) are determined by:

4 $$x_{diameter} = x_{i,{\rm max} } - x_{i,{\rm min} } ,$$

5 $$y_{diameter} = y_{i,{\rm max} } - y_{i,{\rm min} } ,$$

where *x*_i,max_ (*x*_i,min_) is the maximum (minimum) position of the beam diameter in *x*-coordinate direction and *y*_i,max_ (*y*_i,min_) is the maximum (minimum) position of the beam diameter in *y*-coordinate direction.

It should be noted that the centroid algorithm does not need to fit any specific model, therefore, it can also be used for determining the diameters and centers of the Gaussian beam profiles obtained in the second step.

### Detection efficiency homogeneity procedure

The homogeneity of the detection efficiency of the Si-SPAD detector is determined by scanning the active area of the Si-SPAD sensor with a laser beam of a diameter of approx. 10 µm; i.e. focusing the laser beam with an objective lens and scanning it as described in the previous section. However, in this case a monitor detector is used for correcting the possible fluctuation of the laser optical power which may occur during the measurement. The scanning is carried out with a step resolution of 5 µm over the complete active area of the sensor.

It should be noted that for determining the homogeneity of the Si-SPAD detection efficiency only relative measurements are needed. Therefore in this case each signal obtained from the Si-SPAD for each (*x*, *y*) scanning position is normalized to the one obtained when the laser beam is impinging at the center of the sensor active area. That is,

6 $$N(x,y)_{rel} = \frac{N_{x_{i},y_{j}}}{N_{center}}\cdot \frac{s_{{mon}_{center}}}{s_{{mon}_{i,j}}},$$

where $N_{{x_{i} ,y_{j} }}$ is the Si-SPAD counts for the (*x*,*y*)-position, *N*_center_ is the Si-SPAD count rate at the center position *N*_x=0_,_y=0_ and *s*_*mon*_ is the signal of the monitor detector. The homogeneity of the detection efficiency may be defined as the standard deviation of the relative detection efficiency for a defined region.

### Filter transmission procedure

The total filter transmission cannot be measured directly with the standard detector used in our measurement setup, because of the high laser power attenuation required for the calibration of the Si-SPAD detection efficiency. Therefore, a two-step in situ procedure is used, as described in detail in López et al. (2015). Here the transmission of each filter (*T*_F2_ and *T*_F3_) is individually measured, from where the total transmission of the combination of both filters is calculated. Thus, in order to evaluate its associated uncertainty, two filters with lower optical density (higher transmittance) than the filters used in a real calibration of the Si-SPAD detector were used. In this case the combination of both filters can be now measured with a standard detector. The filter transmissions were measured using the integrating sphere with an attached Si-photodiode as detector, thus diminishing effects like back reflection into the setup and stray light. High accuracy translation stages are used for highly reproducible positioning of the filters, thus diminishing inhomogeneity effects. The overall transmission of the filter combination (*T*_combined_) is measured, where both filters are simultaneously positioned in the beam path. For a wavelength dependent investigation, a tunable laser source operating in a wavelength range from 766 to 781 nm is used for the investigation. The deviation between the total filter transmission calculated from the individual filter transmission measurements and the directly determined total filter combination was evaluated by:

7 $$Dev = 1 - \frac{{T_{{F_{2} }} \cdot T_{{F_{3} }} }}{{T_{Combined} }},$$

This deviation is taken as the overall uncertainty contribution of the filter transmission for the determination of the detection efficiency of Si-SPAD detectors, as already described in López et al. (2015).

## Measurement results

### Si-SPAD alignment position

**Fig. 3:**
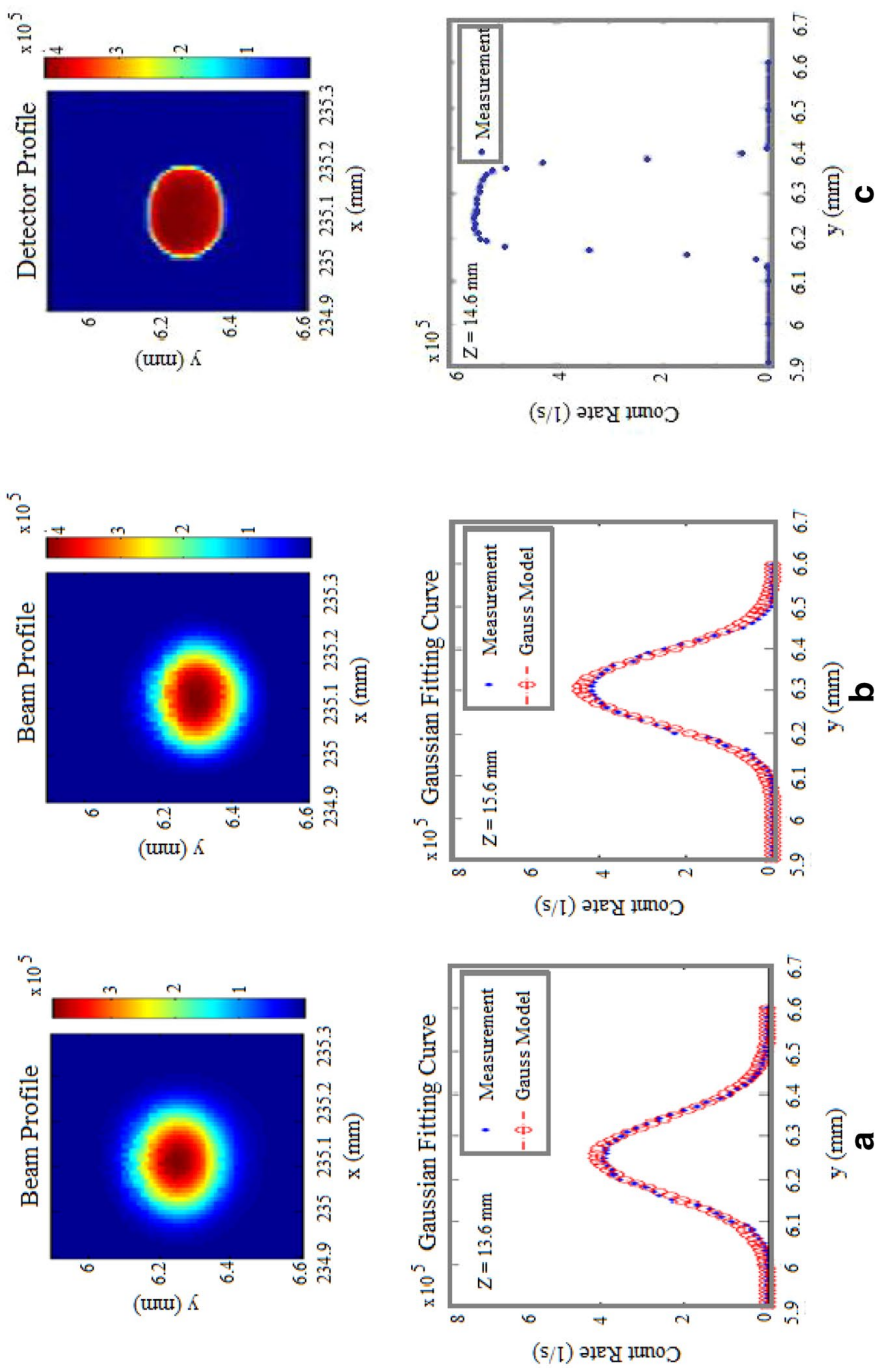
Scanning results for different *z* -positions using the SPCM-AQR (PerkinElmer) detector. **a**, **b** Scan in front of and behind the front focal plane of the objective, i.e. predominantly Gaussian profiles with their fitting curves and **c** scan at the front focal plane of the objective and therefore nearly rectangular profile

Table 1 shows the results for three scans performed using the PerkinElmer SPCM-AQR detector with respect to the different *z*-positions. For the *z*-position of 13.6 mm, i.e. closer to the microscope objective than the focal plane, the scan profile corresponds dominantly to a Gaussian beam profile, see Fig. 3a. The error arising from a fit of the measured scan to a Gaussian curve is only 8.1%. A second *xy*-scan was performed at a *z*-position of 15.6 mm, i.e. further away from the microscope objective focal length, see Fig. 3b. Also here, a dominantly Gaussian profile is measured, with an error from a Gaussian curve of only 8.5%.

**Table 1:** Results obtained from the three scans performed with the SPCM-AQR Si-SPAD detector at different z-positions

Scan number	*z*-position (mm)	*x*-center (mm)	*y*-center (mm)	Diameter (mm)
1	13.6	235.11	6.26	0.35
2	15.6	235.12	6.29	0.35
3	14.6	235.11	6.28	0.20

The values (*x, y* and *z*) obtained from scan Nr. 3 are the final alignment positions where the Si-SPAD must be placed for its calibration. The diameter at this position corresponds approximatly to the diameter (*ϕ*_D_ = 180 μm) of the detector active area

Based on these scans and the determined data, the optimum (*x*,*y*,*z*)-position for the Si-SPAD detector is calculated to *x*_center_ = 235.11 mm, *y*_center_ = 6.28 mm and *z* = 14.6 mm using Eqs. (1)–(3). As expected, the scan profile at this *z*-position corresponds dominantly to a rectangular profile, see Fig. 3c.

**Fig. 4:**
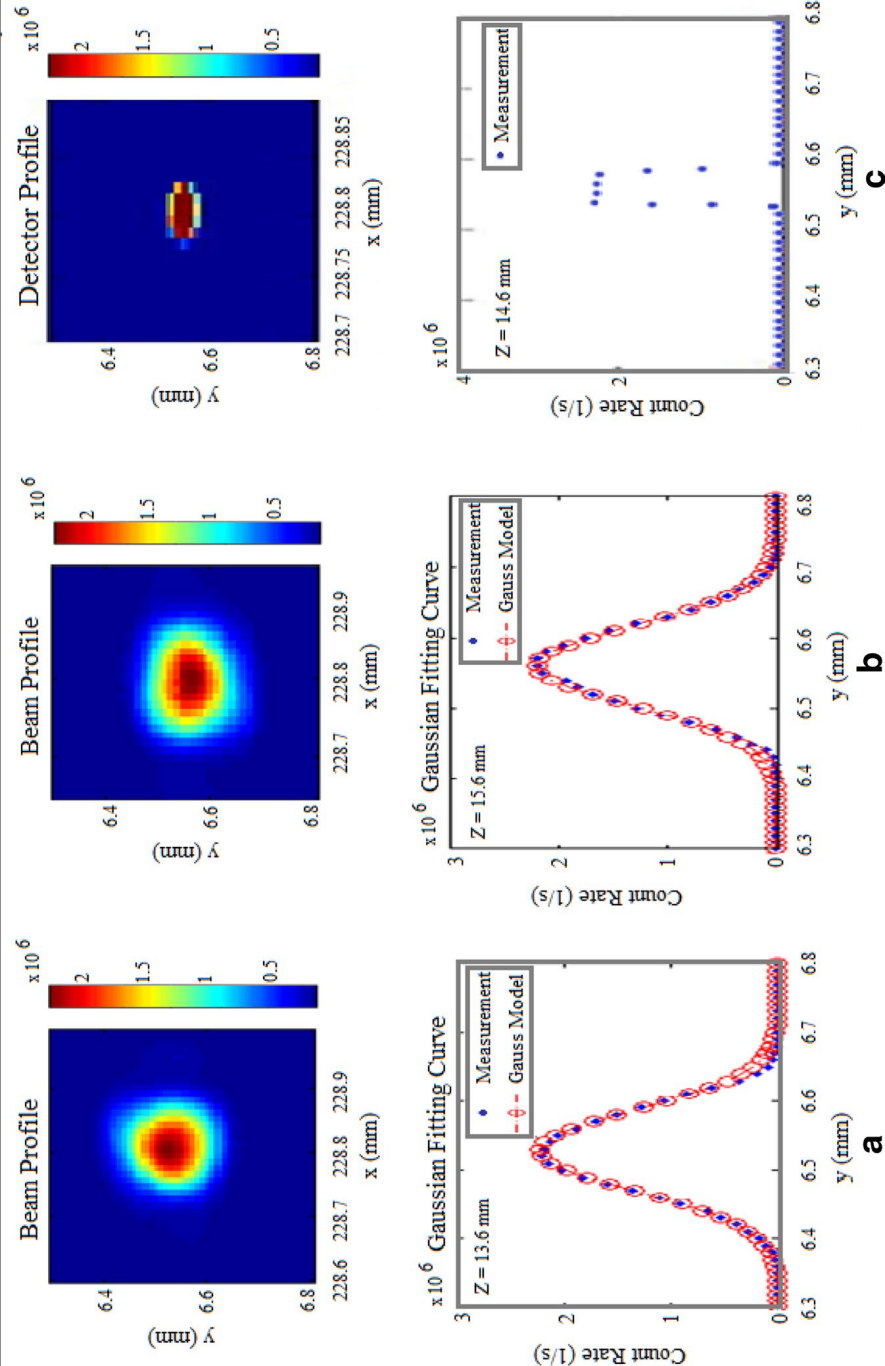
Scanning results for different z -positions using the PDM (Micro Photon Devices) detector. **a**, **b** Scan in front of and behind the front focal plane of the objective, i.e. predominantly Gaussian profiles with their fitting curves and **c** scan at the focal plane of the objective and therefore nearly rectangular profile

The same procedure has been used to determine the optimum (*x*,*y*,*z*)-position of the PDM detector, resulting in *x*_center_ = 228.80 mm, *y*_center_ = 6.55 mm and *z* = 14.6 mm. In Table 2 and Fig. 4 the results for the three scans performed with the PDM detector are summarized.

**Table 2:** Results obtained from the three scans performed with the PDM Si-SPAD detector at different z-positions

Scan number	*z*-position (mm)	*x*-center (mm)	*y*-center (mm)	Diameter (mm)
1	13.6	228.81	6.52	0.23
2	15.6	228.79	6.56	0.23
3	14.6	228.80	6.55	0.04

The values (*x*, *y* and *z*) obtained from scan Nr. 3 are the final alignment positions where the Si-SPAD must be placed for its calibration. The diameter at this position corresponds approximately to the diameter (*ϕ*_D_ = 50 μm) of the detector active area

### Homogeneity

**Fig. 5:**
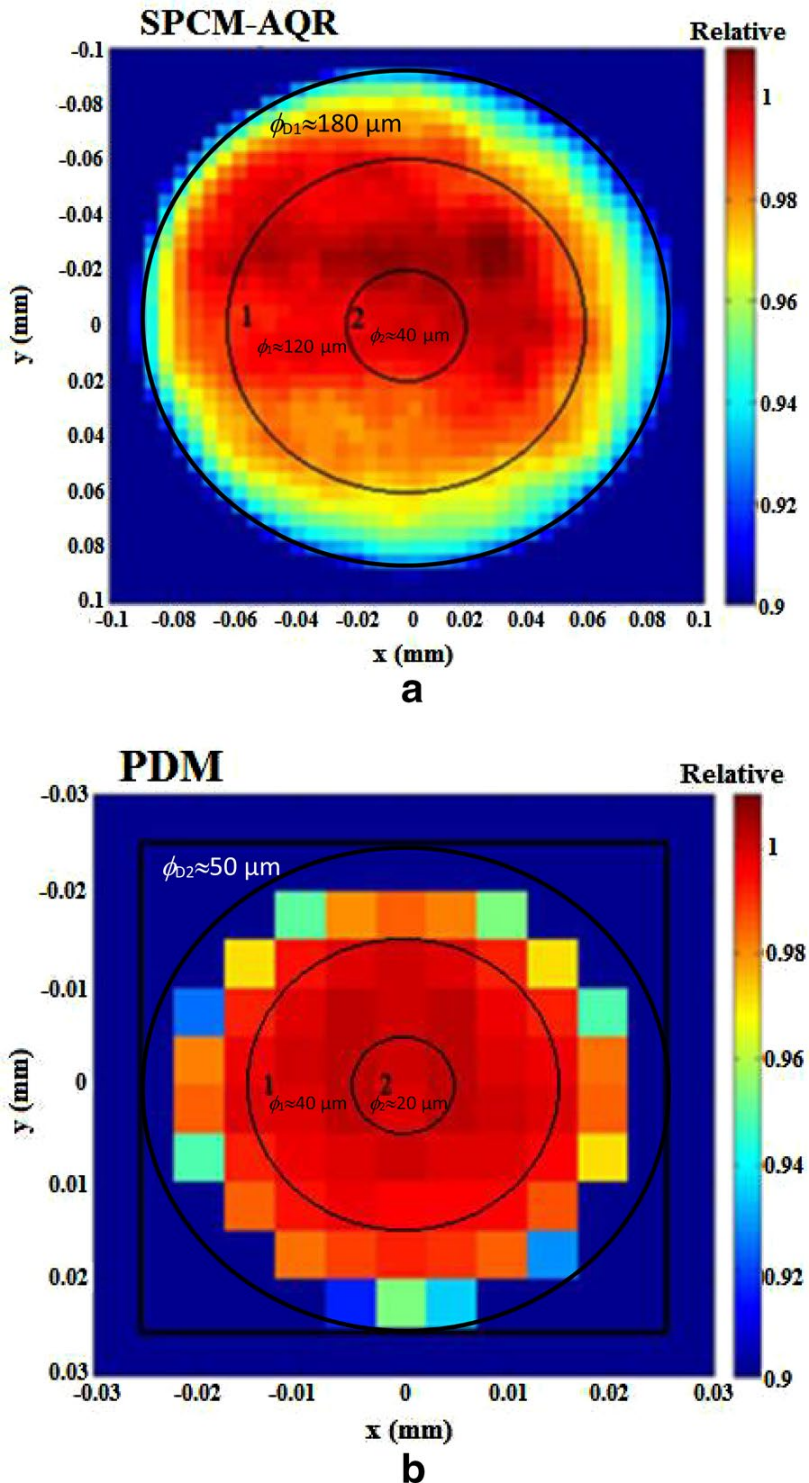
Relative spatial detection efficiency of the Si-SPAD detectors: **a** SPCM-AQR (PerkinElmer) detector, sensor area with diameter *ϕ*_D1_ of approx. 180 µm, determined with a beam diameter *ϕ*_B_ of approx. 10 µm; **b** PDM (Micro Photon Devices detector, sensor area with diameter *ϕ*_D2_ of approx. 50 µm, determined with a beam diameter *ϕ*_B_ of approx. 10 µm. The *circles* labeled with the number 1 and 2 show the regions selected for determining the homogeneity of the detector active area; i.e. the relative standard deviation of the detector spatial responsivity was calculated for these regions

Figure 5a, b show the relative spatial responsivity obtained for the Si-SPAD detector PerkinElmer SPCM-AQR and the Micro Photon Devices PDM detector, respectively. For the analysis of the detector homegeities, the active areas of the detectors were divided in two regions (1, 2) with different diameters. Thus, the homogeneity was evaluated by calculating the standard deviation of the relative detection efficiency for a specific region as described in section—Detection efficiency homogeneity procedure—.

In Fig. 5a, the homogeneity of the detection efficiency of the Si-SPAD PerkinElmer SPCM-AQR, obtained for the mean detection efficiency within the circled region 1 (diameter: 120 µm), is ≤0.85%. However, the homogeneity is improved by selecting smaller regions, i.e. for the region 2 with a diameter of 40 µm the homogeneity obtained is ≤0.3%. Figure 5b shows the homogeneity obtained for the PDM detector. Here the homogeneity obtained for region 1 (diameter: 40 µm) and region 2 (diameter: 20 µm) is ≤2.2% and ≤0.13%, respectively.

**Fig. 6:**
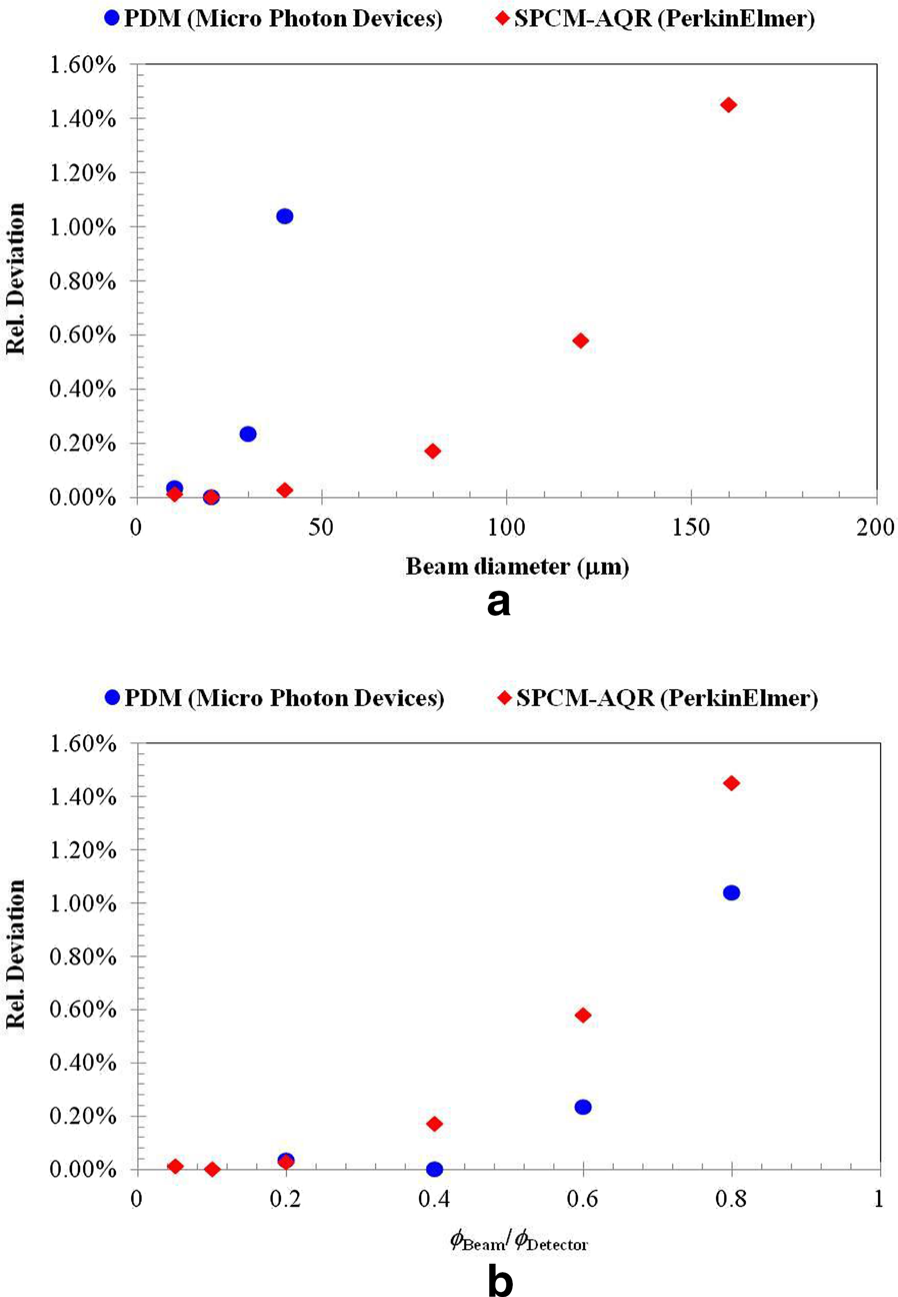
**a** Relative deviation of the detection efficiency of the investigated Si-SPAD detectors for the case when different laser beam diameters are used. The relative deviation is normalized to a beam diameter *ϕ*_B_ of approx. 20 µm; **b** relative deviation of the detection efficiency as a function of the diameter ratio of the beam and the active area of the investigated Si-SPAD detectors

Additionally, the relative deviation of the detection efficiency of the Si-SPAD detectors for different beam diameters is shown in Fig. 6a. For this analysis, the relative deviation of the detection efficiency for different beam diameters has been normalized to the detection efficiency obtained for a beam with a diameter of *ϕ*_B_ = 20 µm impinging on the center of the active areas of the Si-SPAD detectors. It is observed here that the change of the detection efficiency for different beam diameters, originating from the non-perfect homogeneity, is larger for the PDM detector than for the PerkinElmer SPCM-AQR detector. However, this behavior is caused by the smaller active area of the PDM detector. The smaller the active area, the more sensitive is the detection efficiency with respect to an increase in the beam diameter. Therefore, in order to compare the sensitivity of the detection efficiency of these two detectors with different beam diameters, the relative detection efficiency as a function of the ratio between the beam diameter and the active area (sensor diameter) is shown in Fig. 6b. Here it is observed that the detection efficiency of the PerkinElmer SPCM-AQR Si-SPAD detector is more sensitive to changes in the beam diameter. These results clearly show that, depending on the active area of the SPAD sensor, an appropriate laser beam diameter must be used for achieving low measurement uncertainties in the determination of the detection efficiency of a Si-SPAD detector.

### Filter transmission

**Fig. 7:**
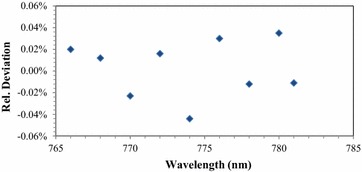
Deviation between the filter transmission measurements calculated according to Eq. (7) for the wavelengths between 766 and 781 nm

The filter transmission measurements were carried out as described in section—Filter transmission procedure—. The deviation between the two filter transmission measurements, i.e. the individual and the combined filter transmission are graphically shown in Fig. 7. The deviations were calculated according to Eq. (7). The maximum deviation is ≤0.05% (at *λ* = 774 nm) within the whole wavelength range from 766 to 781 nm. This maximum deviation is used for the estimation of the uncertainty of the correction factor *F*_filt_. It should be noted, that the observed deviations are in general very small and demonstrate a significant improvement compared to the former setup described in López et al. (2015), where the deviation was about 0.3%. The main reason for this improvement is the use of the integrating sphere instead of the formerly used Si-photodiode. The latter yields a back reflection into the setup, which in turn leads to a slightly higher stray light. This additional stray light leads to a higher signal. In case of the combined measurement with two filters simultaneously positioned in the beam path, this stray light occurs just once. In case of the two individual measurements, this stray light is measured twice, thus a deviation of 0.3% between the measurements occurred. For the integrating sphere, the back reflection is almost completely diminished.

### Detection efficiency and its associated uncertainty

The determination of the detection efficiency of the Si-SPAD detector (Perkin-Elmer SPCM-AQR) and its measurement uncertainty is based on the following equation, see also López et al. (2015):

8 $$\eta = \frac{hc}{\lambda }\frac{{A_{2} A_{3} }}{{A_{1} }}\frac{{Q_{1} Q_{4} }}{{Q_{2} Q_{3} }}s_{Si} F_{filt} ,$$

where *η* is the detection efficiency of the Si-SPAD detector; i.e. the measurand, *h* is the Planck constant, *c* is the speed of light, *λ* is the wavelength, *A*_1_ *, A*_2_ *, A*_3_ are the signal amplification factors of the trans-impedance amplifiers, *Q*_1_ *, Q*_2_ *, Q*_3_ are the ratios of the signal of the Si-photodiode attached to the integrating sphere and the monitor detector signal, *Q*_4_ is the ratio of the count rate and the monitor detector signal, *s*_Si_ is the spectral responsivity of the integrating sphere with the attached Si-photodiode and *F*_filt_ is the factor taking into account the use of two filters.

In the previous work, see López et al. (2015), all components listed above and their associated measurement uncertainties were described in detail. Therefore, in this paper we focus only on those factors and uncertainty components, which were improved. These are in specific the positioning of the Si-SPAD detectors and the filter transmission measurement. The first will in principle lead to smaller statistical (type A) uncertainty contributions, because effects of inhomogeneity will be reduced. The improvement in the filter transmission measurement will reduce one of the major uncertainty components drastically. The maximum deviation measured was 0.05%, therefore this value is used for the overall measurement uncertainty calculation [instead of 0.3% as in López et al. (2015)]. The updated measurement uncertainty budget for this improved measurement setup is shown in Table 3. The improvement for the filter transmission measurements has leads to a practically negligible uncertainty contribution from this uncertainty component. The main contribution comes now from the absolute responsivity calibration of the integrating sphere with the attached detector. Taking into account all uncertainty components, the detection efficiency of the Si-SPAD (Perkin-Elmer SPCM-AQR) detector at 770 nm for a photon rate of approx. 100,000 photons per second and its associated standard measurement uncertainty are:

9a $$\eta_{\text{SPAD}} = 0.5968 \pm 0.001,$$

9b $$\eta_{\text{SPAD}} = 0.5968 \pm 0.16\% .$$

The reproducibility of the detection efficiency measurement obtained for 10 measurements maintaining the same measurement conditions was ≤0.1%.

**Table 3:** Measurement uncertainty budget for determining the detection efficiency of the Si-SPAD detector (Perkin-Elmer SPCM-AQR)

Uncertainty components	Uncertainty (%)
Planck constant, *h*	2.52 × 10^−7^
Speed of light, *c*	0.0
Wavelength, *λ*	0.0075
Amplification factor, *A*_1_	0.0021
Amplification factor, *A*_2_	2.08 × 10^−6^
Amplification factor, *A*_3_	2.08 × 10^−6^
Ratio *V*_1_/*V*_Mon1_, *Q*_1_	0.004
Ratio *V*_2_/*V*_Mon2_, *Q*_2_	0.015
Ratio *V*_3_/*V*_Mon3_, *Q*_3_	0.05
Ratio CR/*V*_MonSPAD_, *Q*_4_	0.036
Spectral responsivity of integrating sphere with Si-diode, *s*_Si_	0.15
Factor for the use of two filters, *F*_filt_	0.05
Combined uncertainty, *u*_*c*_	0.162

## Summary and conclusion

In this paper, the improvement of the measurement setup for the detection efficiency calibration of Si-SPAD detectors was described. These improvements are based on the optimization of the Si-SPAD detector positioning, which is now performed in a completely automated way. Furthermore, the uncertainty contribution due to the filter transmission measurement is practically negligible by using an integrating sphere, which diminishes the back reflection into the measurement setup. The overall relative standard measurement uncertainty for the estimation of the Si-SPAD detection efficiency is now 0.16% instead of 0.3% as in López et al. (2015). However, this value has to be validated by independent measurements and comparisons with other national metrology institutes. The detailed analysis presented in this paper shows the potential for achieving low measurement uncertainties in determining the Si-SPAD detection efficiency even in the low photon flux range.

The homogeneity of the detection efficiency was also investigated. It was shown, that it strongly depends on the beam size impinging on the detector and the regions of its active area. However, the homogeneity can be improved by selecting small regions of the sensor active area, e.g. for a region with diameter of 20 µm, the obtained homogeneity is ≤0.13%. Nevertheless, the low uncertainties are only to be realized for reproducible measurement conditions, i.e. in specific for equal beam sizes and beam shapes and well as for an irradiation of equal active areas of the detector. This, however, will be difficult to obtain when measurements are performed at different national metrology institutes.
